# Quantifying the association between PM_2.5_ air pollution and IQ loss in children: a systematic review and meta-analysis

**DOI:** 10.1186/s12940-024-01122-x

**Published:** 2024-11-18

**Authors:** Naomi C. Alter, Ella M. Whitman, David C. Bellinger, Philip J. Landrigan

**Affiliations:** 1 Boston College Global Observatory on Planetary Health, Boston, MA USA; 2 Department of Neurology, Boston Children’s Hospital and Harvard Medical School, Boston, MA USA; 3 Centre Scientifique de Monaco, Monaco, MC Monaco

**Keywords:** Ambient air pollution, PM_2.5_, IQ, Cognitive function, Children’s environmental health, Burden of disease

## Abstract

**Background:**

A growing body of epidemiologic and toxicologic literature indicates that fine airborne particulate matter (PM_2.5_) pollution is neurotoxic and threatens children’s neurobehavioral development, resulting in reduced cognitive function. Understanding the magnitude of this effect is critical for establishing public health policies that will protect children’s health, preserve human capital, and support societal progress.

**Objective:**

To quantify the association between ambient PM_2.5_ air pollution and loss of cognitive function in children, as measured by Intelligence Quotient (IQ) scores, through a systematic literature review and meta-analysis.

**Methods:**

Following PRISMA guidelines, we conducted a systematic literature search across seven databases: Agricultural and Environmental Science, BIOSIS Citation Index, Embase, GreenFILE, PubMed, Scopus, and Web of Science to identify original scientific studies that investigated the impact of PM_2.5_ exposure during pre-and postnatal periods on IQ loss during childhood. Using data from studies included for final review, we conducted a meta-analysis, using a random effects model to compute a beta coefficient that quantifies the overall effect of PM_2.5_ exposure on Full-Scale IQ (FSIQ), Performance IQ (PIQ), and Verbal IQ (VIQ).

**Findings:**

Of the 1,107 unique publications identified, six studies met the inclusion criteria for final review, representing 4,860 children across three continents (North America, Europe, and Asia). The mean PM_2.5_ concentration across all studies was 30.4 ± 24.4 µg/m^3^. Exposure timing ranged from the prenatal period to mid-childhood. Children were an average of 8.9 years old at the time of cognitive testing. We found that each 1 µg/m^3^ increase in PM_2.5_ concentration is associated with a -0.27 point change in FSIQ (*p* < 0.001), 0.39 point change in PIQ (*p* = 0.003), and -0.24 point change in VIQ (*p* = 0.021).

**Conclusion:**

Through a systematic review and meta-analysis, we identified a statistically significant relationship between increased exposure to PM_2.5_ air pollution and reduced cognitive function in children, with the most pronounced impact on PIQ. This analysis will enable estimation of the burden of adverse neurobehavioral development attributable to PM_2.5_ in pediatric populations and will inform local and global strategies for exposure prevention.

**Supplementary Information:**

The online version contains supplementary material available at 10.1186/s12940-024-01122-x.

## Background

Ambient air pollution is a severe and pervasive hazard to population health. It is of particular concern for the health of children. The World Health Organization (WHO) estimates that 99% of the world’s population breathes air in which levels of fine airborne particulate matter pollution with a mass median diameter of 2.5 µm (μm) or less (PM_2.5_) exceed the WHO guideline of 5 µg/m^3^ air. Due to their extremely small diameter, PM_2.5_ particles can penetrate directly into the brain via the olfactory bulb and deeply into the lungs upon inhalation. The smallest inhaled particles can cross the alveolar-capillary membrane and enter the bloodstream, where they translocate through systemic circulation, inducing oxidative stress and triggering the body’s immune response, leading to persistent inflammation [1–3].

The health consequences of PM_2.5_ pollution exposure include multiple adverse respiratory, cardiovascular, immune, neurological, and neonatal outcomes, including premature mortality [4]. Air pollution was estimated to have caused 6.67 million deaths worldwide in 2019. Approximately two-thirds (4.14 million) of these deaths were attributable to ambient PM_2.5_ pollution. This global burden of disease is inequitable, with an estimated 92% of pollution-related deaths occurring in Low- and Middle-Income Countries (LMICs) [4].

A growing body of literature has examined the neurological effects of PM_2.5_. Studies in adults have identified PM_2.5_ as a risk factor for neurodegenerative conditions, including cognitive impairment, neuronal death, neuroinflammation, and the accumulation of neuropathological markers [5]. In children, a limited but growing number of studies have identified linkages between PM_2.5_ exposure and decreased cognitive performance, quantified by Intelligence Quotient (IQ) loss [6–11]. Other components of air pollution such as sulfur dioxide, nitrogen dioxide, polycyclic aromatic hydrocarbons (PAH), and PM_10_ have also been linked to decreased cognitive performance on verbal and math tests [12, 13]. Two recent reviews have examined associations between air pollution and a suite of child health outcomes, including cognitive and behavioral outcomes [14, 15]; however, neither produced a pooled beta coefficient linking PM_2.5_ exposure and changes in children’s IQ.

Children are uniquely susceptible to PM_2.5_ exposure due to their higher minute ventilation rate, greater oxygen consumption per unit body weight, permeable biological membranes (e.g., blood–brain barrier, airway epithelium), and immature immune/detoxification systems. Children’s vulnerability is further enhanced by the extraordinary complexity of brain development in early life. Damage done to the developing brain in utero and in early postnatal life can result in permanent injury and increase the risk for adverse neurobehavioral outcomes across the life course. At highest risk are children born prematurely and children with pre-existing health conditions.

**Fig. 1:**
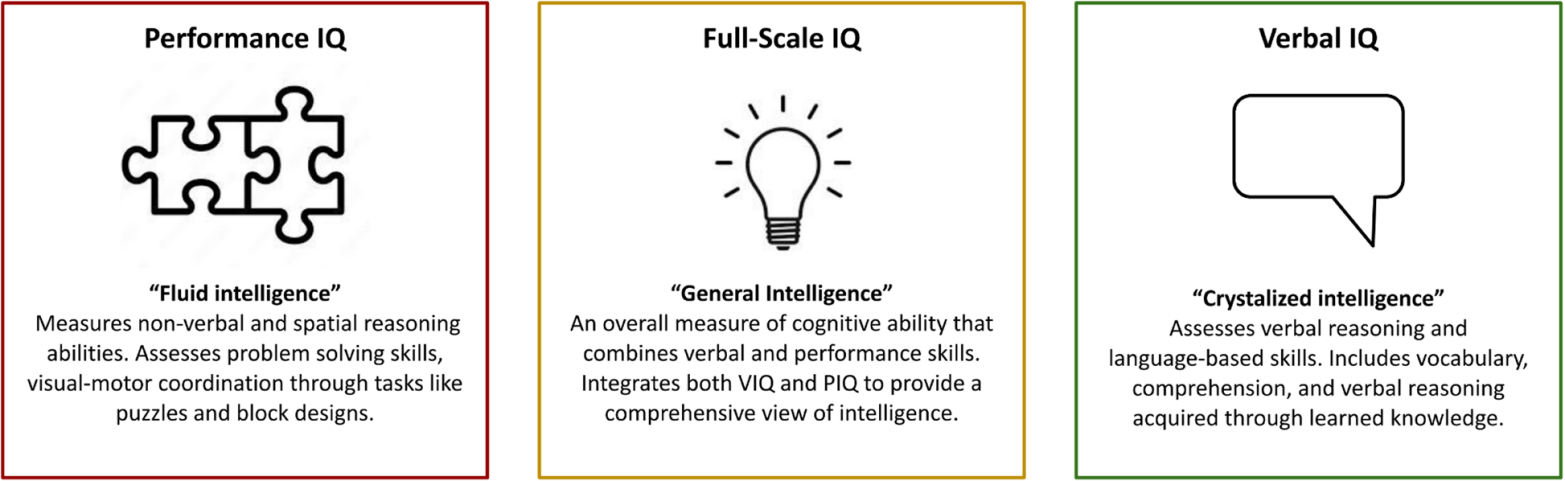
IQ Types Included in Meta Analyses

Until now, a key impediment to quantifying the impact of PM_2.5_ pollution on IQ loss in children has been the lack of an exposure‐response function linking pollution to IQ loss [16]. This study aims to close this knowledge gap and develop concentration‐response functions quantifying the relationship between airborne PM_2.5_ concentrations and IQ loss. Such functions can be applied in future epidemiologic studies and utilized to estimate the global burden of disease attributable to air pollution (Fig. 1).

## Methods

### PECO Statement

The Population, Exposure, Comparator, and Outcomes (PECO) strategy was used to define our research question: *In children aged 0–18 years (Population), how does exposure to higher levels of PM*_*2.5*_* during the prenatal, postnatal, and childhood period (Exposure) compared to lower levels of PM*_*2.5*_* (Comparator) affect neurodevelopment, as measured by IQ scores (Outcome)?*

### Information Sources and Search Strategies

We conducted a systematic literature search following PRISMA guidelines to characterize the correlation between PM_2.5_ exposure and IQ loss in children. The search was conducted across seven databases: Agricultural and Environmental Science, BIOSIS Citation Index, Embase, GreenFILE, PubMed, Scopus, and Web of Science on 27 October 2022 (Table 1). These databases were selected as they covered a broad range of topics related to medicine, public health, and environmental health. Additionally, all the databases met the criterion of being available at Boston College (BC), where the review was conducted. Our search strategy was guided by a BC research librarian. Following the initial systematic review, a second author conducted snowball sampling by reviewing articles that cited the identified studies on 25 September 2023. The newly identified studies were assessed according to the inclusion and exclusion criteria applied in the systematic review to ensure consistency and relevance.

**Table 1:** Search Strategy by Database and Additional Notes

Database	Search Strategy	Notes
Agricultural and Environmental Science	1. (child* OR youth OR kid OR kids OR adolescen* OR school-age* OR baby OR babies OR infan* OR neonat*) AND 2. (intelligen* OR IQ OR “intelligence quotient” OR “cognitive function” OR “intelligence test” OR “intelligence tests”) AND 3. (PM2.5 OR “PM 2.5” OR PM OR “particulate matter” OR “fine particulate matter” OR “air pollution” OR “air pollutant” OR “air pollutants” OR “black carbon” OR particulate* OR aerosol*)	Searched each line as “NOFT” (no full text) Date run: 10-27-2022 Number of records: 522
BIOSIS Citation Index	1. (child* OR youth OR kid OR kids OR adolescen* OR school-age* OR baby OR babies OR infan* OR neonat*) AND 2. (intelligen* OR IQ OR “intelligence quotient” OR “cognitive function” OR “intelligence test” OR “intelligence tests”) AND 3. (PM2.5 OR “PM 2.5” OR PM OR “particulate matter” OR “fine particulate matter” OR “air pollution” OR “air pollutant” OR “air pollutants” OR “black carbon” OR particulate* OR aerosol*)	Searched each line as “TOPIC” Date run: 10-27-2022 Number of records: 133
Embase	1. (child* OR youth OR kid OR kids OR adolescen* OR school-age* OR baby OR babies OR infan* OR neonat*) 2. (intelligen* OR IQ OR “intelligence quotient” OR “cognitive function” OR “intelligence test” OR “intelligence tests”) 3. (PM2.5 OR “PM 2.5” OR PM OR “particulate matter” OR “fine particulate matter” OR “air pollution” OR “air pollutant” OR “air pollutants” OR “black carbon” OR particulate* OR aerosol*) 4. #1 AND #2 AND #3	Searched each line as “EMBASE ONLY” sources Date run: 10-27-2022 Number of records: 360
GreenFILE	1. TI ( (child* OR youth OR kid OR kids OR adolescen* OR school-age* OR baby OR babies OR infan* OR neonat*) ) OR SU ( (child* OR youth OR kid OR kids OR adolescen* OR school-age* OR baby OR babies OR infan* OR neonat*) ) OR AB ( (child* OR youth OR kid OR kids OR adolescen* OR school-age* OR baby OR babies OR infan* OR neonat*) ) OR KW ( (child* OR youth OR kid OR kids OR adolescen* OR school-age* OR baby OR babies OR infan* OR neonat*) ) 2. TI ( (intelligen* OR IQ OR “intelligence quotient” OR “cognitive function” OR “intelligence test” OR “intelligence tests”) ) OR SU ( (intelligen* OR IQ OR “intelligence quotient” OR “cognitive function” OR “intelligence test” OR “intelligence tests”) ) OR AB ( (intelligen* OR IQ OR “intelligence quotient” OR “cognitive function” OR “intelligence test” OR “intelligence tests”) ) OR KW ( (intelligen* OR IQ OR “intelligence quotient” OR “cognitive function” OR “intelligence test” OR “intelligence tests”) ) 3. TI ( (PM2.5 OR “PM 2.5” OR PM OR “particulate matter” OR “fine particulate matter” OR “air pollution” OR “air pollutant” OR “air pollutants” OR “black carbon” OR particulate* OR aerosol*) ) OR SU ( (PM2.5 OR “PM 2.5” OR PM OR “particulate matter” OR “fine particulate matter” OR “air pollution” OR “air pollutant” OR “air pollutants” OR “black carbon” OR particulate* OR aerosol*) ) OR AB ( (PM2.5 OR “PM 2.5” OR PM OR “particulate matter” OR “fine particulate matter” OR “air pollution” OR “air pollutant” OR “air pollutants” OR “black carbon” OR particulate* OR aerosol*) ) OR KW ( (PM2.5 OR “PM 2.5” OR PM OR “particulate matter” OR “fine particulate matter” OR “air pollution” OR “air pollutant” OR “air pollutants” OR “black carbon” OR particulate* OR aerosol*) ) 4. #1 AND #2 AND #3	Date run: 10-27-2022 Number of records: 57
PubMed	1. (child* OR youth OR kid OR kids OR adolescen* OR school-age* OR baby OR babies OR infan* OR neonat*) 2. (intelligen* OR IQ OR “intelligence quotient” OR “cognitive function” OR “intelligence test” OR “intelligence tests”) 3. (PM2.5 OR “PM 2.5” OR PM OR “particulate matter” OR “fine particulate matter” OR “air pollution” OR “air pollutant” OR “air pollutants” OR “black carbon” OR particulate* OR aerosol*) 4. #1 AND #2 AND #3	Date run: 10-27-2022 Number of records: 354
Scopus	1. (child* OR youth OR kid OR kids OR adolescen* OR school-age* OR baby OR babies OR infan* OR neonat*) AND 2. (intelligen* OR IQ OR “intelligence quotient” OR “cognitive function” OR “intelligence test” OR “intelligence tests”) AND 3. (PM2.5 OR “PM 2.5” OR PM OR “particulate matter” OR “fine particulate matter” OR “air pollution” OR “air pollutant” OR “air pollutants” OR “black carbon” OR particulate* OR aerosol*)	Each line was searched within: “Article title”, “Abstract”, and “Keywords” Date run: 10-27-2022 Number of records: 379
Web of Science	1. (child* OR youth OR kid OR kids OR adolescen* OR school-age* OR baby OR babies OR infan* OR neonat*) AND 2. (intelligen* OR IQ OR “intelligence quotient” OR “cognitive function” OR “intelligence test” OR “intelligence tests”) AND 3. (PM2.5 OR “PM 2.5” OR PM OR “particulate matter” OR “fine particulate matter” OR “air pollution” OR “air pollutant” OR “air pollutants” OR “black carbon” OR particulate* OR aerosol*)	Each line was searches as “TOPIC” Date run: 10-27-2022 Number of records: 259

### Eligibility Criteria

Reports identified through these databases were included in this analysis if they met the following criteria:

• Reports of original research (e.g., not review articles)

• Examined human subjects

• Studied children (persons under the age of 18 years)

• Measured PM_2.5_ exposure prenatally and/or postnatally (including early childhood)

• Measured cognitive performance using FSIQ, PIQ, and/or VIQ

• Provided quantitative data on the correlation between PM_2.5_ and IQ

• Represented the most recent report from ongoing studies with multiple publications

### Screening Process

The initial search retrieved 2,064 articles, which were stored in Zotero, a citation-managing software. A total of 957 duplicate reports were removed, resulting in 1,107 unique reports. After screening the titles and abstracts of these reports, 1066 were excluded because they did not meet our eligibility criteria.

**Fig. 2:**
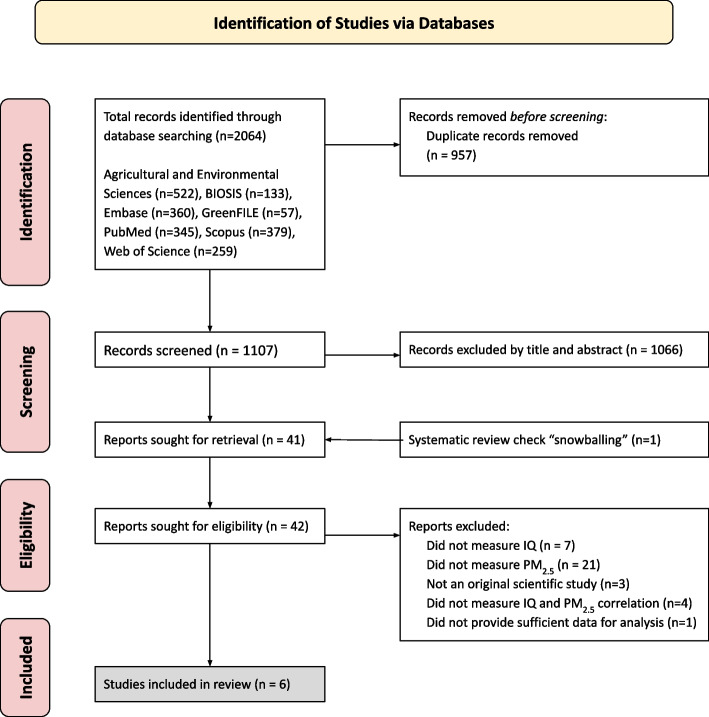
Flow Chart of Literature Search and Selection for Meta-Analysis

The title-abstract screen eliminated articles according to the following criteria: did not involve human subjects (*n* = 25), did not measure PM_2.5_ (*n* = 876), did not measure IQ (*n* = 153), was not an original research report (*n* = 9), or a more recent report was available from the same ongoing study (*n* = 2). After excluding these records, 41 records were retrieved for full-text screening, and an additional study was added following snowball sampling (*n* = 42). Full-text screening excluded an additional 36 articles because they did not meet the eligibility criteria. Reasons for exclusion at this level were that the study did not measure PM_2.5_ (*n* = 21), did not measure IQ (*n* = 7), was not an original scientific study (*n* = 3), did not report a correlation between PM_2.5_ exposure and IQ (*n* = 4), or did not provide sufficient data for analysis (*n* = 1). In sum, this process identified six studies for inclusion in the final review and used a pooled beta coefficient using a random effect meta-analysis (Figs. 1 and 2).

### Data Collection and Preprocessing

Data from the six studies included for final review were compiled in Table 2. Exposure details, including the exposure window (e.g., prenatal or postnatal), exposure location, and exposure measurement, were compiled in Table 3. Cohort details, including size, location, name, and recruitment strategy, were compiled in Tables 3 and 4.

**Table 2:** Studies Included for Final Review

Study	Study Type	IQ Type	IQ Measurement	Original Study Effect Measurement Type	Relevant Calculation	Beta coefficient (points lost per 1µg/m^3^ increase in PM_2.5_ concentration	Notes on Model Adjustments
Harris et al. (2015) [7]							
	Cohort	VIQ	Kaufman Brief Intelligence Test (KBIT-2)	The mean difference in IQ points associated with an increase in PM_2.5_	${\mathrm\beta}_{VIQ}=\frac{-1.0\;points}{2.5\mathrm\mu g/m^3}$	-0.40	Estimates were based on a “minimal model” which was adjusted for child sex and age at cognitive testing. Change in IQ was reported for each 2.5μg/m^3^ increase in PM_2.5_. values were divided by 2.5 to yield IQ points lost per 1μg/m^3^ increase in PM_2.5_)
		PIQ			${\mathrm\beta}_{\mathit P\mathit I\mathit Q}=\frac{-0.4\;points}{2.5\mathrm\mu g/\mathrm m^3}$	-0.16	
Ni et al. (2022) [11]							
	Cohort	FSIQ	Stanford-Binet Intelligence Scales, Fifth Edition (SB-5) and the Wechsler Intelligence Scale for Children, Fifth Edition (WISC-V), and the Wechsler Preschool & Primary Scale of Intelligence, Fourth Edition (WPPSI-IV)	The beta coefficient of the change in IQ points per 2μg/m^3^ increase in PM_2.5_	${\mathrm\beta}_{FSIQ}=\frac{-0.26\;points}{2\mathrm\mu g/m^3}$	-0.13	Estimates based on Model 2, the “primary model”, which controlled for child sex, age, study site, child race, maternal education, log-transformed region, inflation-adjusted household income, household members, an interaction between household members and income, material status, maternal age at delivery, birth order, pregnancy smoking, pregnancy alcohol consumption, maternal depression, maternal IQ, child second-hand smoke exposure, and Child Opportunity Index (domains of educational and economic opportunity) in the corresponding window of PM_2.5_ exposure. Ni and colleagues originally reported IQ points lost per 2μg/m^3^ increase in PM_2.5_ values were divided by 2 to yield IQ points lost per 1μg/m^3^ increase in PM_2.5_) $\mathrm\beta=\frac{\triangle\;IQ\mathit\;points}{\triangle\;{PM}_{\mathit2\mathit.\mathit5}\;(linear\mathit\;coef\mathit\;ficient)}$
Porta et al. (2016) [9]							
	Cohort	FSIQ	Wechsler Intelligence Scale for Children-III edition (WISC-III)	The beta coefficient of the change in IQ points per 10μg/m^3^ increase in PM_2.5_	${\mathrm\beta}_{FSIQ}=\frac{-1.9\;points}{10\mathrm\mu g/m^{\mathit3}}$	-0.19	All models were adjusted for gender, child age (in months) at the time of cognitive test, maternal and paternal educational level, socioeconomic index at birth, maternal age at delivery, maternal smoking during pregnancy, number of older siblings, psychologist who administered the cognitive test, and inversely weighted for the probability of participation at baseline and at the followup (to reduce attrition bias). Porta and colleagues originally reported IQ points lost per 10μg/m^3^ increase in PM_2.5_ values were divided by 10 to yield IQ points lost per 1μg/m^3^ increase in PM_2.5_ $\mathrm\beta=\frac{\triangle\;IQ\mathit\;points}{\triangle\;PM_{2.5}\mathit\;(linear\mathit\;coef\mathit\;ficient)}$
		PIQ			${\mathrm\beta}_{PIQ}=\frac{-4.1\;points}{10\mathrm\mu g/m^3}$	-0.41	
		VIQ			${\mathrm\beta}_{VIQ}=\frac{-0.44\;points}{10\mathrm\mu g/m^{\mathit3}}$	-0.044	
Seifi et al. (2021) [12]							
	Cross-sectional	FSIQ	Raymond B. Cattle Scale I-A	The difference in average IQ of children residing in three geographical locations with low, intermediate, and high M_2.5_ exposure levels.	$\mathrm\beta=\frac{\left(-21.33\right)\left(8.23\right)+\left(-2.33\right)\left(-0.87\right)+\left(23.67\right)\left(-8.37\right)}{\left(-21.33\right)^2+\left(-2.33\right)^2+\left(23.67\right)^2}$	-0.36	All models were adjusted for age, gender, economic conditions, maternal education, and type of delivery. Seifi and colleagues reported the difference in average IQ points from three different groups of children residing in low, intermediate, and high PM_2.5_ exposures. PM_2.5_ was found to be 38.97±16.87 μg/m^3^, 58±23.94 μg/m^3^, and 84.18±31.32 μg/m^3^, respectively. The IQ of children in the area with high pollution was 7.48 lower than that in moderate pollution and 16.628 lower than that in the area with low pollution. To re-express these data as a linear relationship, we created a scatter plot of IQ change vs. PM_2.5_ levels for the three groups on a graph and calculated a line of best fit using Stata statistical software. $\mathrm\beta=\frac{\sum(x_{\mathit i}\mathit-x)(y_{\mathit i}\mathit-y)}{\sum{(x_{\mathit i}\mathit-x)}^2}$ Where is the mean PM_2.5_ levels for each group and is the IQ differences for each group
Sun et al. (2023) [8]							
	Cohort	FSIQ	Wechsler Abbreviated Scale of Intelligence (WASI)	Beta coefficient representing the change in IQ per 5μg/m3 increase in PM2.5 exposure	${\mathrm\beta}_{FSIQ}=\frac{-1.34\;points}{5\mathrm\mu g/m^{\mathit3}}$	-0.27	Models adjusted for maternal factors (age, BMI before pregnancy, IQ, parity, education, intake of folic acid in early pregnancy, depression in early pregnancy), paternal factors (education), gestational weeks, and trimester-specific temperature and humidity. Sun and colleagues originally reported IQ points lost per 5μg/m^3^ increase in PM_2.5_ values were divided by 5 to yield IQ points lost per 1μg/m^3^ increase in PM_2.5_ $\mathrm\beta=\frac{\triangle\;IQ\mathit\;points}{\triangle\;PM_{\mathit2\mathit.\mathit5}\;(linear\mathit\;coef\mathit\;ficient)}$
Wang et al. (2017) [10]							
		FSIQ	Wechsler Abbreviated Scale of Intelligence (WASI)	The mean difference in IQ points associated with interquartile increases in PM_2.5_	${\mathrm\beta}_{FSIQ}=\frac{-2.00\mathit\;points}{21.13-16.09\mathrm\mu g/m^{\mathit3}}$	-0.40	Estimates based on “Adjusted Model IId” which incorporated both within-family (random intercepts and slopes for PM_2.5_ effects) and within-individual (random intercepts) covariances. These models were adjusted incrementally for two sets of covariates: (1) individual and family characteristics—age (continuous or dichotomized into pre-/early-adolescence vs. emerging adulthood), sex, race/ethnicity, family SES, and parental cognitive abilities; and (2) neighborhood characteristics—neighborhood SES, greenspace (1000m radius, 1 year preceding the test), traffic density (300m radius), and parent-reported neighborhood quality.
		PIQ			${\mathrm\beta}_{PIQ}=\frac{-3.08\;points}{21.13-16.09\mathrm\mu g/m^{\mathit3}}$	-0.61	Wang et al. reported PM2.5 concentrations in interquartile ranges: Q1 (2.14-16.08μg/m3 ), Q2: (6.09 - 18.67 μg/m^3^), Q3: (18.68 - 21.13 μg/m^3^), Q4: (21.14 - 25.36 μg/m^3^). To re-express the effect estimate as a slope of the relationship between PM_2.5_ concentration and IQ, we divided the reported IQ points lost by the interquartile range for PM_2.5_ (i.e. the 75th minus the 25th percentile of the distribution), which was identified to be 5.04 μg/m^3^ $\mathrm\beta=\frac{\triangle\;IQ\mathit\;points}{\triangle\;PM_{\mathit2\mathit.\mathit5}(Q\mathit4\mathit/Q\mathit3\mathit\;boundary\mathit-Q\mathit2\mathit/Q\mathit1\mathit\;boundary)}$

**Table 3:** Exposure Details

**Study**	**ExposureWindow**	**PM2.5 mean **± **SD **(μg/m3 )	**Exposure Location**	**Exposure Measurement**
**Harris et al. (2015) **[7]	Prenatal (third trimester)	12.3 ± 2.6	Residential addresses of the birthing parent reported at study visits and on annual questionnaires were geocoded and spatially joined to pollution estimate models.	Satellite aerosol optical depth measurements at the 10x10 km grid scale for years 2000-2010 from the Moderate Resolution Imaging Spectroradiometer aboard the Earth Observing System satellites. Additional inputs to PM_2.5_ concentration measurements from the US Environmental Protection Agency and Interagency Monitoring of Protected Visual Environment networks, along with data on area point sources of PM_2.5_, land use, locations of major roads, and meteorology
**Porta et al. (2016) **[9]	Postnatal	19.5 ± 2.2	Participants' residential addresses at birth and all of their following residential addresses through the age of cognitive assessment were geocoded and spatially joined to pollution estimate models.	Land use regression models developed within the European Study of Cohorts for Air Pollution Effects (ESCAPE). Rome particulate matter levels were measured at 20 sites from 2010-2011 over three separate 2-week periods: cold (January-March), warm (June-September), and intermediate (April-June) seasons. Results from the tree measurements at each site were averaged, adjusting for temporal variations using centrally located background reference sites, which took measurements for an entire year (cross-validation R^2^ = 0.79).
**Wang et al. (2017) **[10]	Postnatal	13.7 ± 6.7	Residential addresses for families were prospectively collected through self-reports every 2-3 years. Addresses were geocoded to match residencies by exact parcel locations or specific street segments of participating families	Daily PM_2.5_ concentrations were obtained from the US Environmental Protection Agency Technology Transfer Network for the years 2000-2014. A spatial-temporal model based on the measured PM_2.5_ concentration was constructed which had high consistency (R^2^ = 0.74-0.79) to estimate the monthly average for each subject’s geocoded residential location.
**Seifi et al. (2021) **[12]	Childhood	Low 39.0± 16.9 Intermediate 58.0 ± 23.9 High 84.2 ± 32.2	Three low-privileged geographic locations (A, B, C) were selected to conduct monitoring. An equal number of participants from the three respective locations were randomly selected to undergo IQ testing	Real-time measurements of PM_2.5_ mass concentrations were provided from environmental dust monitors based on an optical scattering method. Indoor and outdoor exposure was simultaneously measured using direct reading equipment
**Ni et al. (2022) **[11]	Prenatal (pregnancy average)	8.75 ± 2.0	Residential addresses were collected from participants at enrollment and updated at each subsequent point of contact	Point-based PM_2.5_ levels were estimated from a spatial-temporal model on a 2-week scale. This model used monitoring data from regulatory networks, further enhanced with air pollution measurements from intensive research cohort-specific monitors. A geographic information system was used to identify covariates representing land use characters that could reflect spatial variability in air pollution distributions and the dimension-reduced regression covariates were obtained using partial least squares from more than 400 geographic variables.
**Sun et al. (2023) **[8]	Prenatal (first trimester)	38.8 ± 6.2	Geographical coordinates of participants based on birthing parent’s residential addresses. During follow-up visits, migration was taken into consideration by averaging exposure levels if multiple residences were reported.	Satellite-based modeling and aerosol optical depth retrieval and GEOS-Chem simulations. Ground measurements from approximately 1000 monitors were used for cross-validation. Predictions were highly consistent with the real-time measurements (R^2^ = 0.78)

The mean exposure to PM_2.5_ across six studies included in the final review was 30.4 24.4 µg/m^3^

**Table 4:** Cohort Details

Study	Cohort Size	Location	Cohort Name (if applicable)	Recruitment Strategy	Age at Cognitive Testing
Harris et al. (2015) [7]	1109	Massachusetts, USA	Project Viva Cohort	Pregnant people-child pairs enrolled during 1999-2002 at birthing individual’s initial prenatal visits (median, 9.9 weeks of gestation) at eight locations of Atrius Harvard Vanguard Medical Associates, a multi-subspecialty group practice in urban and suburban eastern Massachusetts.	8 years
Porta et al. (2016) [9]	474	Rome, Italy	Gene and Environment Prospective Study on Infancy in Italy (GASPII)	Newborns enrolled at two large obstetric hospitals in Rome in 2003-2004. The eligible population included infants born to women ages 18 years or older and residents of one of the five local health districts in the city.	7 years
Wang et al. (2017) [10]	1085	California, USA	Risk Factors for Antisocial Behavioral (RFAB) twin study	Families were recruited from Los Angeles and surrounding counties, with the resulting sample representative of a socio-economically-diverse multi-ethnic population residing in the greater Los Angeles areas.	9-11 and 18-20 years
Seifi et al. (2021) [12]	369	Buscher providence, Iran	N/A	Children selected from schools in three low-privileged areas in Bushehr province, southern Iran between 2019-2020.	6-8 years
Ni et al. (2022) [11]	1311	California, New York, Minnesota, Washington, USA	Environmental Influences on Child Health Outcomes (ECHO)^a^	***Conditions Affecting Neurocognitive Development and Learning in Early Childhood (CANDLE)*** From 2006-2011 birthing individuals considered eligible if 16-40 years of age, had medically low-risk singleton pregnancies, and planned to deliver in a participating study hospital. ***The Infant Development and Environment Study (TIDES)*** From 2010-2012 recruitment commenced in academic medical centers in four cities: San Francisco, California; Rochester, New York; Minneapolis, Minnesota; and Seattle, Washington. Pregnant individuals in the first trimester were considered eligible if over 18 years of age, were English-speaking, and planned to deliver at a participating study hospital. ***Global Alliance to Prevent Prematurity and Stillbirths (GAPS)*** From 2017-2020 birthing parent-child dyads were recruited from Seattle and Yakima, Washington if they had consented to prenatal questionnaire data and biospecimen collection.	4-6 years
Sun et al. (2023) [8]	512	Shanghai, China	Shanghai-Minhang Birth Cohort	Pregnant people who underwent their first prenatal examination at 12-16 weeks of gestation in the Minhang Maternal and Child Health Hospital in 2012.	6 years

The six studies included for the final review represent a total of 4860 children across three continents (North America, Europe, and Asia). The average age at cognitive testing was 8.9 years

^a^The ECHO cohort was created by pooling three individual prospective cohort studies: Conditions Affecting Neurocognitive Development and Learning in Early Childhood (CANDLE), The Infant Development and Environment Study (TIDES), and Global Alliance to Prevent Prematurity and ssssStillbirths (GAPS)

### Data Synthesis

To obtain comparable values for each of our two variables, results from included studies were expressed as a linear slope (IQ points lost per 1ug/m3 increase in PM_2.5_ concentration) when reported otherwise. Relevant calculations for individual studies are available in Table 2. Data standardization conformed to the following formulas:

**Table:** 

For studies that reported changes in IQ per interquartile range of PM_2.5_ exposure:	${\beta }=\frac{\Delta IQ points}{\Delta { PM}_{2.5} (Q4/Q3 boundary - Q2/Q1 boundary)}$
For studies that reported changes in IQ per X linear coefficient increase in PM_2.5_:	${\beta }=\frac{\Delta IQ points}{\Delta { PM}_{2.5} (linear coefficient)}$
For studies that reported mean differences in IQ points for multiple PM_2.5_ exposure groups:	$\beta =\frac{\sum ({x}_{i}-x)({y}_{i}-y)}{\sum ({x}_{i}-x{)}^{2}}$ Where $x$i are the mean PM_2.5_ levels for each group, and ${y}_{i}$ is the IQ differences for each group

### Statistical Analysis

All meta-analytic techniques were carried out using the package metafor in R statistical software (v4.4.1; R Core Team 2024), an open-source platform. To calculate a beta coefficient relating PM_2.5_ and IQ, we performed a meta-analysis using a random-effects model to account for between-study variability and provide a more generalized estimate of the effect size, given the heterogeneity across studies. The beta coefficient (overall effect size) represents the pooled effect across all included studies, accounting for both within- and between-study variability. Compared to a fixed-effects model, the random-effects model provides a more conservative estimate of the effect size, as it incorporates between-study heterogeneity.

### Risk of *Bias* (RoB)

To evaluate potential biases that may affect the validity of study findings, we used a standardized Risk of Bias (RoB) framework, assessing six domains: selection bias, confounding bias, measurement of exposure, outcome assessment bias, attrition bias, and reporting bias. If each domain in a study was ranked as “low,” its overall Rob was determined as “low”. If the study had domains ranked as “low" and “moderate,” its overall Rob was determined as “moderate”. Details are specified in Supplemental Table S1.

### Heterogeneity

Given that our three meta-analyses included a small number of studies, we assessed for heterogeneity both quantitatively and qualitatively. Using the metafor package in RStudio we calculated the I^2^ statistic, which represents the proportion of variation in effect sizes due to heterogeneity rather than chance, tau (the estimated standard deviation of the actual effect sizes e.g. between-study variability), and p-value (heterogeneity) from the Cochran’s Q-test (Supplemental Table S3). Following this quantitative analysis, we visually inspected the forest plots, paying attention to the degree of overlap of confidence intervals and position of study estimates (e.g., if they were clustered or staggered).

## Results

Six epidemiological studies (five cohort and one cross-sectional) were included in our final analysis (Table 2), representing data from 4,860 children across three continents (North America, Europe, and Asia). The mean level of PM_2.5_ exposure was 30.4 ± 24.4 μg/m^3^ (Table 3), and the mean age at IQ testing was 8.9 years. (Table 4) Without exception, each study reported a negative association between PM_2.5_ exposure and children’s cognitive function. We present findings by order of study publication date.

In the first study, Harris et al. 2015 [7] examined the impact of prenatal exposure to PM_2.5_ on PIQ and VIQ scores of children from the Project Viva Cohort (Massachusetts, USA) using a cohort study design. Pregnant individuals were recruited during prenatal visits (median 9.9 weeks of gestation) at eight locations for Atrius Harvard Vanguard Medical Associates, a multi-subspecialty group practice in urban and suburban eastern Massachusetts. Exposure assessment was conducted by spatially joining geocoded residential addresses of the birthing parent (reported at study visits and on annual questionnaires) to PM_2.5_ models that used satellite aerosol optical depth measurements. The average PM_2.5_ concentration was 12.3 ± 2.6 µg/m^3^ (Table 3). IQ was assessed using the Kaufman-Brief Intelligence Test, Second Edition (KBIT-2) for 1109 children at eight years of age. Estimates on the impact of PM_2.5_ on child IQ were based on a “minimally adjusted model,” accounting for child sex and age at cognitive tests. The authors found that an increase of 2.5 μg/m^3^ in PM_2.5_ concentration in the year before testing was associated with a -0.4 (-1.8, 1.0) point change in PIQ and a -1.0 (-2.2, 0.2) change in VIQ. To re-express this relationship as a beta coefficient, we divided the change IQ points by 2.5. Thus, each 1 µg/m^3^ increase in PM_2.5_ concentration was associated with a change in -0.16 PIQ points and -0.40 VIQ points (Table 2).

In the second study, Porta et al. 2016 [9] examined postnatal exposure to PM_2.5_ and children’s FSIQ, PIQ, and VIQ scores from the Gene and Environment Prospective Study on Infancy in Italy (GASPII) using a cohort study design. Newborns born at two large obstetric hospitals in Rome, Italy to individuals over the age 18 from 2003–2004 were enrolled. Exposure assessment was conducted by spatially joining geocoded residential addresses of participants (as reported at study visits on questionnaires) to PM_2.5_ estimates generated from land-use regression models developed within the European Study of Cohorts for Air Pollution (ESCAPE). The average PM_2.5_ concentration was 19.5 ± 2.2 µg/m^3^ (Table 3). IQ was assessed using the Wechsler Intelligence Scale for Children, Third Edition (WISC-III) for 474 children at seven years of age. Estimates modeling the impact of PM_2.5_ on IQ were adjusted for child age, gender, maternal and paternal education level, socioeconomic index at birth, maternal age at delivery, maternal smoking during pregnancy, number of older siblings, and the psychologist who administered the test. Attrition bias was reduced by inversely weighting for the probability of participation at baseline and follow-up. The authors determined that for each 10 μg/m^3^ increase in PM_2.5_ exposure during pregnancy, there was a change in -1.9 (-7.9, 4.1) FSIQ points, -0.44 (-5.5, 6.4) change in VIQ points, and -4.1 (-3.4, 1.2) change in PIQ points. We divided IQ points lost by a coefficient of 10 to re-express the reported relationship in IQ points lost per 1 μg/m^3^ increase in PM_2.5_. Thus, each 1 µg/m^3^ increase in PM_2.5_ concentration was associated with a change in -0.19 FSIQ points, -0.04 points, -0.04 VIQ points, and -0.41 PIQ points (Table 2).

In the third study, Wang et al. 2017 [10] examined the impact of postnatal exposure to PM_2.5_ on the FSIQ and PIQ scores of children from the Risk Factors for Antisocial Behavioral (RFAB) twin study (California, USA) using a cohort study design. Families were recruited from Los Angeles and surrounding counties, generating a socio-economically diverse, multi-ethnic population. Exposure assessment was conducted by spatially joining geocoded residential addresses (identified through self-reports every 2–3 years) to PM_2.5_ estimates generated from a spatial–temporal model from the United States Environmental Protection Agency Technology Transfer Network. The average PM_2.5_ concentration was 13.7 ± 6.7 µg/m^3^ (Table 3). IQ was assessed using the Wechsler Abbreviated Scale of Intelligence (WASI) for 1085 children aged 9–11 and 18–20. Estimates on the impact of PM_2.5_ on child IQ were based on “Adjusted Model IId,” which adjusts for individual/family characteristics (e.g., age, sex, race/ethnicity, socioeconomic status, parental cognitive abilities) and neighborhood characteristics (e.g., greenspace, traffic density, and parent-reported neighborhood quality). The authors divided the sample into four quartiles according to PM_2.5_ exposure: in quartile 1, PM_2.5_ exposures ranged from 2.14–16.08 μg/m^3^; in quartile 2, (16.09–18.67 μg/m^3^), quartile 3, (8.66–21.12 μg/m^3^), and quartile 4, (21.14–25.36 μg/m^3^). For each interquartile increase in PM_2.5_, there was a -2.00 (-4.84, 0.24) point change in FSIQ, -3.08 (0.12—6.04) point change in PIQ, and -1.42 (-4.48, 1.64) change in VIQ. To re-express this relationship as a beta coefficient, we divided the reported IQ points lost by the interquartile range for PM_2.5_ (i.e., the 75th minus the 25th percentile of the distribution). From this calculation, we determined that each 1 μg/m^3^ increase in PM_2.5_ concentration is associated with a change in -0.40 FSIQ points, -0.61 PIQ points, and -0.28 VIQ points (Table 2).

In the fourth study, Seifi et al. 2021 [12] examined the impact of childhood exposure to PM_2.5_ on the FSIQ scores of children residing in Bushehr province, Iran, using a cross-sectional study design. Children were randomly selected from schools in three low-privileged geographic areas between 2019 and 2020. Exposure assessment was conducted using real-time measurements of PM_2.5_ mass concentrations from environmental dust monitors based on an optical scattering method. Indoor and outdoor exposures were simultaneously measured using direct reading equipment. The average PM_2.5_ concentrations were 39.0 $\pm$ 16.9 µg/m^3^ for the low-exposure group, 58.0 $\pm$ 23.9 µg/m^3^ for the intermediate-exposure group, and 84.2 $\pm$ 32.2 µg/m3 for the high-exposure group (Table 3). IQ was assessed using the Raymond B. Cattle Scale I-A for 369 children at six to eight years of age. Estimates on the impact of PM_2.5_ on child IQ adjusted for e adjusted for age, gender, economic conditions, maternal education, and type of delivery. The authors determined that children's IQ in the area with high pollution was 7.48, lower than that in moderate pollution, and 16.628, lower than that in the region with low pollution. To re-express these data as a linear relationship, we created a scatter plot of IQ change vs. PM_2.5_ levels for the three respective exposure groups and fitted a regression line using Stata statistical software. Using this method, each 1 µg/m^3^ increase in PM_2.5_ concentration was associated with a -0.36 change in FSIQ points (Table 2).

In the fifth study, Ni et al. 2022 [11] examined the impact of prenatal exposure to PM_2.5_ on FSIQ of children from the Environmental Influences on Child Health Outcomes (ECHO) Cohort, with representation from California, New York, Minnesota, and Washington USA. The ECHO Cohort was created by pooling participant data from three individual prospective cohort studies, detailed in Table 4. Exposure assessment was conducted by spatially joining geocoded residential addresses of participants (collected at enrollment and updated at each subsequent point of contact) to PM_2.5_ estimates from spatial–temporal models using data reported on a two-week scale. Additional cohort-specific monitors enhanced these measurements. The average PM_2.5_ concentration was 8.75 $\pm$ 2.0 µg/m^3^ (Table 3). IQ was assessed using the Stanford-Binet Intelligence Scales, Fifth Edition (SB-5), the Wechsler Intelligence Scale for Children, Fifth Edition (WISC-V), and the Wechsler Preschool & Primary Scale of Intelligence, Fourth Edition (WPPSI-IV) for 1311 children at four to six years of age. Estimates on the impact of PM_2.5_ on child IQ were based on the “primary model,” which controlled for child sex, age, study site, child race, maternal education, log-transformed region, inflation-adjusted household income, household members, an interaction between household members and income, material status, maternal age at delivery, birth order, pregnancy smoking, pregnancy alcohol consumption, maternal depression, maternal IQ, child second-hand smoke exposure, and Child Opportunity Index (domains of educational and economic opportunity) in the corresponding window of PM_2.5_ exposure. The authors determined that a 2 µg/m3 increase in PM_2.5_ during pregnancy was associated with − 0.26 (− 1.53, 1.01) points. To re-express this relationship as a beta coefficient, we divided the change IQ points by 2. Thus, each 1 µg/m^3^ increase in PM_2.5_ concentration was associated with -0.13 FSIQ points (Table 2).

In the sixth study, Sun et al. 2023 [8] assessed the impact of prenatal exposure to PM_2.5_ on FSIQ of children from the Shanghai-Minhang Birth Cohort (Shanghai, CN) using a cohort study design. Pregnant individuals who underwent their first prenatal examination at 12–16 weeks of gestation in the Minhang Maternal and Child Health Hospital in 2012 were enrolled. Exposure assessment was conducted by spatially joining geocoded residential addresses of the birthing parent (reported during enrollment and follow-up visits) to PM_2.5_ estimates developed using satellite-based monitoring and aerosol optical depth retrieval. Ground measurements from approximately 1000 monitors were used for cross-validation, which was reported to be consistent with real-time measurements (R^2^ = 0.78). The average PM_2.5_ concentration was 38.8 $\pm$ 6.2 µg/m^3^ (Table 3). IQ was assessed using the Wechsler Abbreviated Scale of Intelligence (WASI) for 512 children at six years of age. Estimates on the impact of PM_2.5_ on child IQ adjusted for maternal factors (age, BMI before pregnancy, IQ, parity, education, intake of folic acid in early pregnancy, depression in early pregnancy), paternal factors (education), gestational weeks, and trimester-specific temperature and humidity. The authors reported a -1.34 (− 2.71, 0.04) change in FSIQ points for every 5 µg/m3 increase in PM_2.5_ during the first trimester of pregnancy. To re-express this relationship as a beta coefficient, we divided the change IQ points by 5. Thus, each 1 µg/m^3^ increase in PM_2.5_ concentration was associated with -0.27 FSIQ points (Table 2).

Pooling data from the studies included for final review, our meta-analyses indicated a significant negative association between PM_2.5_ and each IQ domain. Specifically, a 1 µg/m^3^ increase in ambient PM_2.5_ is associated with a -0.27 (-0.37, -0.17) point change in FSIQ (*p* < 0.001), a -0.39 (-0.65, -0.14) point change in PIQ (*p* = 0.003), and a -0.24 (-0.45, -0.04) point change in VIQ (*p* = 0.021) (Tables 5, 6 and 7). Forest plots are detailed in Tables 5, 6 and 7.

**Table 5:**
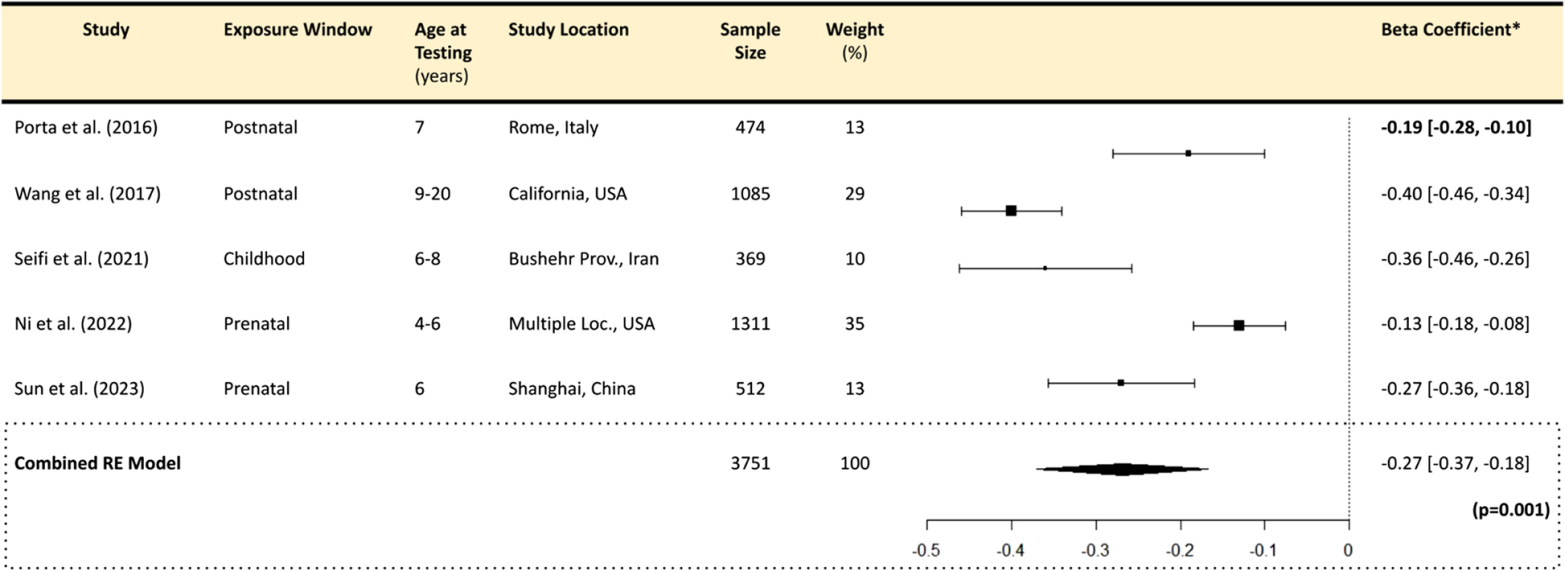
PM_2.5_ and Full-Scale IQ

Forest plot showing effect of PM_2.5_ exposure on FSIQ using a random effects model. A significant overall effect size of -0.27 (-0.37, -0.18) FSIQ points lost per 1µg/m³ increase in PM_2.5_ (*p* = 0.001) was observed. Plot presents point estimates and 95% confidence intervals for each study included in the meta-analysis. Individual studies are represented by squares (point estimates) and horizontal lines (confidence intervals). Pooled effect estimate is indicated by a diamond at the bottom, with its width representing the confidence interval

*IQ Points lost per 1µg/m^3^ increase in PM_2.5_ All meta-analyses generated using a Random Effects model in R Statistical Software (v4.4.1)

**Table 6:**
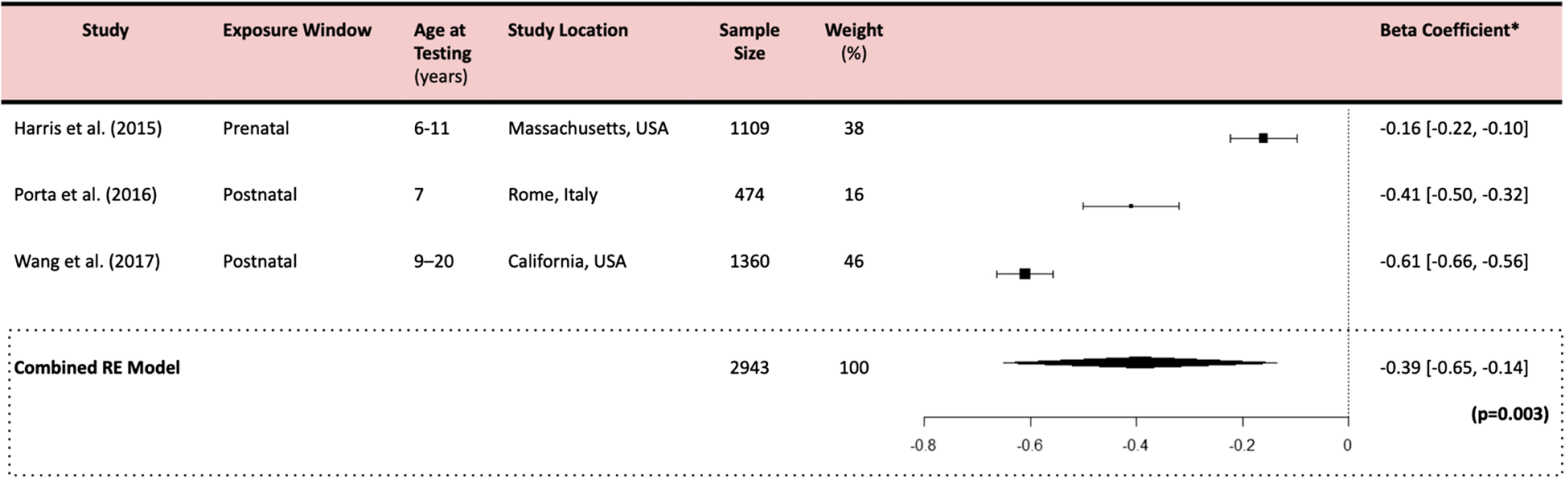
PM_2.5_ and Performance IQ

Forest plot showing effect of PM_2.5_exposure on PIQ using a random effects model. A significant overall effect size of -0.39 (-0.65, -0.14) PIQ points lost per 1µg/m³ increase in PM_2.5_ (*p* = 0.003) was observed. Plot presents point estimates and 95% confidence intervals for each study included in the meta-analysis. Individual studies are represented by squares (point estimates) and horizontal lines (confidence intervals). Pooled effect estimate is indicated by a diamond at the bottom, with its width representing the confidence interval

*IQ Points lost per 1µg/m^3^ increase in PM_2.5_ All meta-analyses generated using a Random Effects model in R Statistical Software (v4.4.1)

**Table 7:**
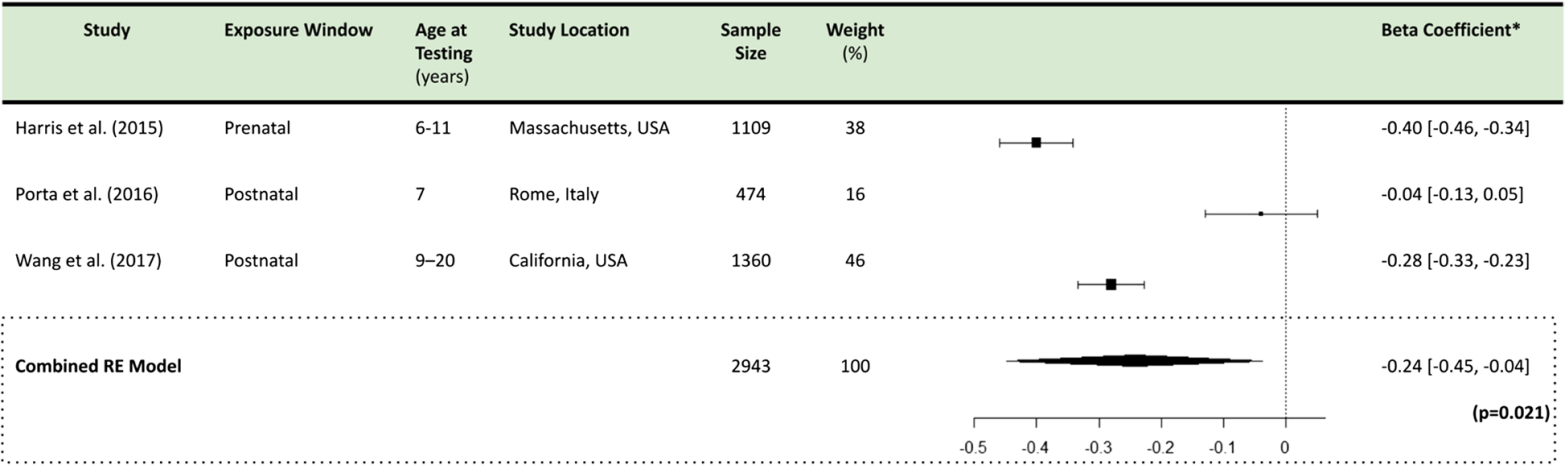
PM_2.5_ and Verbal IQ

Forest plot showing effect of PM_2.5_ exposure on VIQ using a random effects model. A significant overall effect size of -0.24 (-0.45, -0.04) VIQ points lost per 1µg/m³ increase in PM_2.5_ (*p* = 0.021) was observed. Plot presents point estimates and 95% confidence intervals for each study included in the meta-analysis. Individual studies are represented by squares (point estimates) and horizontal lines (confidence intervals). Pooled effect estimate is indicated by a diamond at the bottom, with its width representing the confidence interval

*IQ Points lost per 1µg/m^3^ increase in PM_2.5_ All meta-analyses generated using a Random Effects model in R Statistical Software (v4.4.1)

Overall, we determined that there was a high degree of heterogeneity among our studies. Through our quantitative analysis, the I^2^ statistic was high (> 88%), and Cochran’s Q test of heterogeneity was significant (*p* < 0.05) for all meta-analyses (Supplemental Table S3). Qualitatively, we observed a moderate degree of overlap in the confidence intervals and a narrow spread of the effect sizes around the pooled estimates in our FSIQ model, suggesting moderate heterogeneity (Table 5), while a slight overlap of the confidence intervals for our PIQ and VIQ models (Tables 6 and 7), suggesting high heterogeneity.

## Discussion

To our knowledge, this is the first study to quantify the correlation between PM_2.5_ air pollution exposure and decreased cognitive function in children based on a systematic review of the world’s literature. All six of the studies included in our analysis consistently found that prenatal and childhood exposures to PM_2.5_ pollution are associated with declines in verbal and nonverbal cognitive abilities, as measured by IQ loss. Our findings, which include data from geographically, socially, economically, and culturally diverse populations, indicate that PIQ is the component of cognitive function most profoundly affected by PM_2.5_. PIQ is more severely affected than either VIQ or FSIQ, suggesting that it is an especially sensitive indicator of brain injury caused by adverse environmental exposures.

Children’s communication and language skills—the “crystallized” cognitive abilities reflected by VIQ —may be more resilient to adverse environmental exposures such as PM_2.5_ pollution due to the ubiquitous nature and importance of daily communication and social interaction, as opposed to PIQ, which reflects non-verbal, more “fluid” cognitive abilities such as the ability to reason and to solve novel problems and is contingent on adequate sensory input and rich play environments. Differences in critical periods of brain development may also help to elucidate the differential impact of PM_2.5_ on PIQ. Thus, experience-dependent synapse formation peaks around two months of age for the visual cortex [17], a brain structure supporting PIQ, whereas the receptive language/speech production area (critical for VIQ) peaks later, around eight months of age (Supplemental Figure S1). It is possible that children are more susceptible to PM_2.5_ exposure in the early postnatal period when the development of brain regions associated with PIQ is most rapid.

Loss of non-verbal cognitive function, as measured by PIQ loss, has significant implications for children’s health, development, and future life accomplishments. Long-term follow-up studies of children who suffered IQ loss from early-life exposures to neurotoxicants other than PM_2.5_ pollution have found linkages to a range of developmental and behavioral deficits. These include a shortened attention span, compromised reading and math abilities, reduced control over impulsive and aggressive behaviors, and increased rates of school failure [18]. Longer-term ramifications include increased risks of juvenile delinquency, criminal behavior, and incarceration [19–21]. Additional neurobehavioral consequences of early-life exposures to PM_2.5_ pollution include increased incidence of behavioral abnormalities, such as autism spectrum disorder (ASD) and attention-deficit/hyperactivity disorder (ADHD) [14, 15, 22–28].

Population-wide reduction in mean cognitive capacity by as little as 5 IQ points results in a more than 50% decrease in the number of children with superior intelligence (IQ above 130) and a corresponding increase in the number of children with IQ scores below 70. Such a loss of cognitive capacity in a population represents a massive erosion of human capital, reduces a society’s future leadership potential, and threatens national survival [29]. At the same time, the significant increase in the number of children with reduced cognitive capacities imposes substantial economic and social burdens on societies by reducing the lifelong productivity of future generations and increasing the need for remedial education and custodial care. For these reasons, and given the widespread nature of children’s exposure to excessive levels of PM_2.5_ air pollution, our finding that early-life PM_2.5_ exposure is associated with concentration-related loss of cognitive function in children has significant economic and policy implications [30].

The high degree of heterogeneity we observed (both quantitatively and qualitatively) across the studies we examined was expected as the studies included for final review used different methodologies to model air pollution exposure (Table 3) and examined neurobehavioral outcomes of children at various ages in diverse geographical locations (Table 4). Further, differential access to opportunities for educational and social engagement may have mediated cognitive outcomes, obscuring the direct effects of PM_2.5_. For these reasons, our findings should be interpreted cautiously and reexamined as additional data become available.

Our findings are consistent with an expanding body of literature on the harmful impacts of pre- and postnatal exposures to PM_2.5_ air pollution on children’s neurobehavioral development [14, 15]. Because many millions of children across the globe are exposed to PM_2.5_ pollution, the aggregate losses in cognitive function resulting from these exposures have the potential to be as large or more prominent than those caused by other exposures to other widespread neurotoxicants. A study of Full-Scale IQ (FSIQ) losses in American children less than 5 years of age reported that lead was responsible for an aggregate loss of 23,000,000 FSIQ points, methylmercury for a loss of 285,000 FSIQ points, and organophosphate pesticides for a loss of 17,000,000 FSIQ points [31].

Our study has several limitations. First, our derivation of a concentration–response function relating PM_2.5_ concentration to IQ loss in children is based on only six studies. This scarcity of available data underscores the early stage of research on the neurobehavioral consequences of early-life exposure to air pollution. Moreover, only one author screened all studies included in this analysis, which may introduce bias in the selection process. To mitigate this limitation, future research should consider employing multiple reviewers to ensure a more rigorous and unbiased screening.

Another limitation is that we were not able to assess the potentially synergistic impacts of air pollution and other neurotoxicants in children’s development. Although all of the studies in our analysis included comprehensive evaluations of potential confounding from demographic and socioeconomic variables, none formally evaluated the possibility of other adverse environmental exposures exacerbating the adverse impacts of increased PM_2.5_ on children’s IQ scores. Further, our models do not account for potential non-linear trends, an essential consideration for geographic locations with pollution exposures outside our study range. Mounting evidence from other toxicological exposures (e.g., endocrine-disrupting chemicals from plastic additives) suggests that non-monotonic dose–response relationships may be common. We lack the data to determine whether the concentration–response functions we derived for IQ loss at PM_2.5_ concentrations are linear, supralinear, attenuated, or flat at higher PM_2.5_ concentrations. Estimating IQ losses in children with higher exposures will necessitate either direct study or extrapolations that incorporate assumptions about the shape of the concentration–response function at those exposures.

Yet another limitation of our study is that it could not account for differences in the chemical constituents of PM_2.5_ pollution that may occur in different places around the world. For example, biomass burning in wildfires produces PM_2.5_ with higher concentrations of PAHs as opposed to more complete combustion processes such as vehicle emissions. Such variability could modify the concentration–response function. However, in the absence of studies that parse out the relative contributions of different constituents of PM_2.5_, the assumption of equitoxicity has been widely used in epidemiologic studies of the health effects of air pollution, including the Global Burden of Disease (GBD) study [32–34].

We note that IQ loss in children caused by harmful environmental exposures such as PM_2.5_ pollution is not included in the Global Burden of Disease (GBD) calculations unless exposures are so severe that they result in IQ scores below 70 (the criterion for diagnosing a child as having an “intellectual disability”) [32, 34]. The consequence of this unfortunate omission is that most cognitive impairments associated with toxic environmental exposures are not counted in the GBD study. This impedes the estimation of the actual population health impacts and human capital losses caused by air pollution and other neurotoxicants. For the many countries that utilize GBD findings to guide priority setting and resource allocation in public health, this omission underestimates the adverse impacts of air pollution on children’s cognitive function and results in opportunities for prevention being lost. We encourage future research initiatives undertaken in partnership with the GBD study collaborators to address this information gap and improve the ability of the GBD study to guide preventive policy in pediatrics [32, 33].

Future epidemiological studies should seek to diversify their patient populations and geographical locations, as we were unable to identify studies matching our search criterion that represented populations in South America, Africa, or Oceania. We note that each study included for the final review used proximal estimates to determine children’s PM_2.5_ exposure (e.g., geographical information systems, land-use regression models, satellite imagery). While effective and efficient for population-level cohorts, continued research should seek to quantify exposure at the individual level (e.g., biomarkers and wearable devices) to increase sensitivity and precision when generating exposure–response functions. In the setting of unprecedented planetary changes, we stress the need to evaluate the impact of PM_2.5_ on children’s neurodevelopment with other adverse co-exposures, such as extreme heat, stress from displacement/migration, altered nutrient availability/quality, and increased vector-borne disease.

Lastly, since children’s brains are experiencing rapid periods of tightly organized growth and development, it is necessary to track IQ over time to characterize neurodevelopmental trajectories. For example, the difference between IQ scores among children of high SES versus those of low SES was tripled at age 16, when compared to their difference at age two in a British cohort [35] – demonstrating that adverse events/exposures during the early years of childhood exacerbate existing health inequities, but also serve as opportunities for resiliency in children’s neurodevelopment.

## Conclusion

Our combined effect estimates, based on data generated from six epidemiological studies representing children from three continents (North America, Europe, and Asia), use standard coefficients, supporting a negative impact of PM_2.5_ exposure on children’s neurocognitive development, as measured by IQ scores. Though relatively small, the estimated effect sizes for FSIQ, PIQ, and VIQ are of significant public health importance, considering the lifelong effects of adverse neurodevelopmental on children’s health, well-being, and human capital and the wide extent of children’s exposure to levels of PM_2.5_ air pollution that exceed WHO guidelines. Substantial gains in economic productivity have occurred in countries that reduced airborne lead pollution by removing lead from gasoline [30]. Similar benefits may be expected to result from sustained reductions in PM_2.5_ pollution. Developing an exposure-response function linking PM_2.5_ concentration to IQ loss provides the means for quantifying these benefits and translating them into public policy.

## Supplementary Information

Supplementary Material 1.

## Data Availability

No datasets were generated or analysed during the current study.

## Abbreviations

IQ: Intelligence Quotient
FSIQ: Full-Scale Intelligence Quotient
PIQ: Performance Intelligence Quotient
VIQ: Verbal Intelligence Quotient
PM: Particulate Matter
PM_10_: Particulate Matter Air Pollution 10 Micrometers in Diameter
PM_2.5_: Particulate Matter Air Pollution 2.5 Micrometers in Diameter
SDI: Socio-demographic Index
WHO: World Health Organization
LMIC: Low or Middle-Income Country
