# Supplementary material for: Quantifying the association between PM2.5 air pollution and IQ loss in children: a systematic review and meta-analysis
Source: Environ Health. 2024 Nov 18;23:101. doi: 10.1186/s12940-024-01122-x (PMC11572473; doi:10.1186/s12940-024-01122-x)
Supplement: Supplementary file 1 — Supplementary Material 1. [file 12940_2024_1122_MOESM1_ESM.pdf]

Supplemental Material

Table S1. Risk of Bias (RoB) Assessment

Study	Selection Bias	Confounding Bias	Measurement of Exposure	Outcome Assessment Bias	Attrition Bias	Reporting Bias	Overall ROB
**Harris et al. (2015)**	**Low:** Study sample was recruited from a multi-specialty group practice that cares for ethnically and racially diverse patients across the spectrum of socioeconomic status in urban and suburban communities of eastern Massachusetts. Characteristics of included participants were generally similar to those of participants excluded due to missing exposure or outcome data, although excluded participants had somewhat lower birth weight.	**Low:** Models adjusted for a variety of biological and socioeconomic factors.	**Low:** Geocoded subject addresses were joined with satellite aerosol optical depth measurements at the 10x10 grid scale.	**Low:** Standardized Kaufman Brief Intelligence Test (KBIT-2) IQ assessments were administered to subjects by trained professionals.	**Low:** >75% retention from enrollment to IQ assessment.	**Low:** All pre-specified outcomes reported.	**Low**
**Porta et al. (2016)**	**Moderate:** Subjects were recruited from six maternity units in five Italian cities (Florence, Rome, Trieste, Turin, and Viareggio) in the Gene and Environment Prospective Study in Italy (GASPII). A higher proportion of well-educated birthing parent-child dyads participated in the study compared to lower-educated dyads.	**Low:** Models adjusted for several demographic and socioeconomic factors.	**Low**: Geocoded addresses were joined with land-use regression models developed to estimate annual PM2.5 using data from twenty air monitors; 90% of individuals remained at the same address for the duration of the study.	**Low:** Standardized Wechsler Intelligence Scale for Children-III (WISC-III) IQ assessments were administered to subjects by trained professionals.	**Moderate:** 67% retention from enrollment to IQ assessment.	**Low:** All pre-specified outcomes reported.	**Moderate**
**Wang et al. (2017)**	**Moderate:** Subjects recruited from the University of Southern California (USC) Risk Factors for Antisocial Behavior twin study. The subject sample was representative of a socio-economically diverse, multi-ethnic population residing in Los Angeles and surrounding communities. Subjects tested with higher IQ scores were more likely to follow up, but their IQ did not differ from those tested during the emerging adulthood period.	**Low:** Models were adjusted for individual, family, and neighborhood characteristics (see Table 1 for further details).	**Low:** Geocoded addresses were joined with spatiotemporal models of monthly PM2.5 estimates.	**Low:** Standardized Wechsler Abbreviated Scale of Intelligence (WASI) IQ tests were administered to subjects by trained professionals.	**Low:** >75% retention from enrollment to IQ assessment.	**Low:** All pre-specified outcomes reported.	**Moderate**
**Seifi et al. (2021)**	**Low:** Subjects were recruited from schools in the Busher province in southern Iran. Demographic and socioeconomic factors were consistent across all three levels (low, intermediate, high) of $PM_{2.5}$ exposure.	**Low:** Models were adjusted for several demographic and socioeconomic factors.	**Low:** Real-time measurements of mass concentrations of PM2.5 were provided by direct reading devices using environmental dust monitors based on optical scattering methods	**Low:** Standardized Raymond B. Cattell scale I-A IQ assessments were administered to subjects by trained professionals.	**Low:** 100% retention from enrollment to IQ assessment (since cross-sectional study design)	**Low:** All pre-specified outcomes reported.	**Low**
**Ni et al. (2022)**	**Low:** Study sample was derived from the ECHO-PATHWAYS Consortium (comprising three prospective epidemiological cohorts) spanning geographically diverse regions of the United States. The analytic sample (those assessed for IQ) was similar to the overall sample of participants at enrollment.	**Low:** Models adjusted for several indicators of child and family characteristics, including multilevel social determinants.	**Low:** Geocoded addresses were joined with spatial-temporal models that generated PM2.5 estimates on a two-week scale.	**Low:** Standardized Stanford-Binet Intelligence Scales, Fifth Edition (SB-5) and the Wechsler Intelligence Scale for Children, Fifth Edition (WISC-V), and the Wechsler Preschool & Primary Scale of Intelligence, Fourth Edition (WPPSI-IV) IQ tests were administered to subjects by trained professionals.	**Moderate:** >66% retention from enrollment to IQ assessment.	**Low:** All pre-specified outcomes reported.	**Moderate**
**Sun et al. (2023)**	**Moderate:** Study sample was recruited from the Shanghai-Minhang Birth Cohort. Compared with the excluded children, those included for IQ testing had older mothers and higher maternal and paternal educational levels. No other characteristics showed statistically significant differences between the two groups.	**Low:** Models adjusted for parental, medical, socioeconomic, and lifestyle factors.	**Low:** Geocoded addresses were joined with PM2.5 estimates from satellite imagery. Aerosol optical depth retrieval was used in the modeling procedure.	**Low:** Standardized Wechsler Intelligence Scale for Children, 4th edition (WISC-IV, Chinese version) IQ assessments were administered to subjects by trained professionals. This version of the WISC-IV was adapted for Chinese children to address language and cultural differences.	**Low:** >75% retention from enrollment to IQ assessment.	**Low:** All pre-specified outcomes reported.	**Moderate**

RoB was assessed for the six studies included for final review. Two studies, Harris et al (2015), Seifi et al. (2021) were determined to have a low overall RoB while three studies (Porta et al. 2016), Wang et al. (2017), and Sun et al. (2023) were determined to have a moderate overall Rob.

Table S2. RoB Assessment Domains

Bias Domain	Description
**Selection Bias**	Evaluates how participants were selected and whether the sample is representative of the target population.
**Confounding Bias**	Assesses the adequacy of control for confounding variables that could affect the outcomes.
**Measurement of Exposure**	Considers the reliability and validity of exposure assessment methods.
**Outcome Assessment Bias**	Examines how outcomes were measured and whether there was any bias in the assessment process.
**Attrition Bias**	Look at the completeness of follow-up and whether the reasons for dropout were related to the outcome.
**Reporting Bias**	Evaluates whether all pre-specified outcomes were reported and whether there was selective reporting of results.

Adapted from Sterne et al.(1)

Table S3. Summary Statistics of Meta-Analyses Examining $PM_{2.5}$ and Child IQ Loss

	**Combined Estimate**			**Heterogeneity**				
**IQ Measurement**	**Beta Coefficient***	**p-value**	**Total Sample Size**	**I^2**	**Tau**	**Q**	**P-value (heterogeneity)**	**Notes/comments: visual inspection of forest plot (Figures 4a-c)**
FSIQ (Table 5a)	-0.27 [-0.60, 0.04]	0.001	3382	89.34%	0.11	49.5	<0.01	Moderate degree of overlap in the confidence intervals and a narrow spread of the effect sizes around the pooled estimates suggested moderate heterogeneity.
PIQ (Table 5b)	-0.42 [-0.79, -0.05]	0.003	2794	97.74%	0.05	114	<0.01	Low degree of overlap in the confidence intervals and a moderate spread of the effect sizes around the pooled estimates suggested high heterogeneity.
VIQ (Table 5c)	-0.28 [-0.30, -0.03]	0.021	2794	96.64%	0.22	43.1	<0.01	Low degree of overlap in the confidence intervals and a moderate spread of the effect sizes around the pooled estimates suggested high heterogeneity.

* IQ Points lost per 1ug/m3 increase in $PM_{2.5}$ concentration. All meta-analyses were generated using a Random Effect model in RStudio. All beta coefficients derived from combined data were significant ($p<0.05$). I^2 represents the proportion of variation in effect sizes due to heterogeneity rather than chance. Tau (τ) represents the estimated standard deviation of the true effect sizes (between-study variability). The p-value from Cochran's Q-test confirmed significant heterogeneity. Notes/Comments were detailed after visual inspection of the forest plots displayed in Figures 4a-c.

Table S3. Mechanisms of $PM_{2.5}$-Induced Cognitive Impairment in Children: Impacts on Performance and Verbal IQ with Considerations of Unique Vulnerabilities

Mechanistic Pathway	Description	Citation	Impact on PIQ	Impact on VIQ	Children's Unique Vulnerability
**Inflammation** 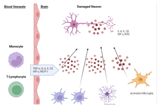	$PM_{2.5}$ exposure initiates systemic inflammation by activating microglia, increasing the levels of pro-inflammatory cytokines such as IL-6 and TNF-α. This inflammation can affect brain regions such as the prefrontal cortex, crucial for executive functions, memory, and cognitive processing (2,3,4).	Gómez-Budia et al. 2020<br>Song et al. 2022<br>Calderón-Garcidueñas et al. 2023	Chronic inflammation can impair cognitive functions that involve executive control, memory, and attention, leading to lower PIQ scores in tasks requiring complex problem-solving and spatial reasoning.	Although inflammation predominantly affects executive functions, prolonged exposure may lead to broader cognitive deficits, indirectly impacting language processing abilities.	Children have developing immune systems that may overreact to pollutants, causing heightened inflammatory responses. The developing brain is more sensitive to inflammatory mediators, which can disrupt neurodevelopment and cognitive functions.
**Oxidative Stress Induction** 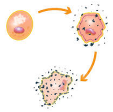	$PM_{2.5}$ contains metals and organic compounds that generate reactive oxygen species (ROS) in the respiratory epithelium. These ROS can enter systemic circulation, cross the blood-brain barrier and cause oxidative damage to neural cells (4,5).	Shang et al. 2023<br>Calderón-Garcidueñas et al. 2023	Oxidative stress can lead to neuronal damage in brain areas responsible for executive function and spatial awareness, impairing performance on tasks involving problem-solving and abstract reasoning.	While oxidative stress primarily affects cognitive functions related to processing and memory, secondary impacts on brain health can affect language acquisition and comprehension over time.	Children's brains have high metabolic rates (relative to their body mass) and have underdevolaped immune and renal systems making them more susceptible to oxidative damage. The high plasticity of their brains means that oxidative stress can lead to more significant long-term cognitive impairments.
**Epigenetic Changes** 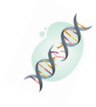	miRNAs are a crucial target of epigenetic damage caused by air pollution, which play important roles in gene transcription. Their mature forms contribute to the degradation of the mRNAs and thus transcriptional and post-transcriptional repression (6).	Gavito-Covarrubias et al. 2024	Epigenetic factors related to neural plasticity may play a role in enhancing or inhibiting cognitive flexibility, affecting performance IQ which requires adapting to novel situations.	Epigenetic modifications can affect genes involved in language acquisition and processing. For instance, factors influencing neural development and synaptic plasticity may enhance abilities in vocabulary and grammar.	Rapid, tightly coordinated developmental processes in neural development during the prenatal, postnatal, childhood, and adolescent periods are contingent on controlled gene expression. When disrupted, children's growth is altered at the cellular and physiological level. These changes can shape their future developmental trajectories and can even be passed on to subsequscent generations.
**Synaptic Disruption** 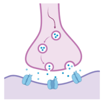	$PM_{2.5}$ exposure during critical developmental periods can disrupt the normal development of brain structures involved in cognition and language. Disruption of neurogenesis and synaptic development can lead to abnormalities in brain regions such as the temporal lobe, parietal lobe, hippocampus and prefrontal cortex (7).	Han et al 2024	Disrupted brain development can impair cognitive functions that are crucial for performance IQ, including executive functioning, working memory, and spatial reasoning abilities.	Disruptions in early brain development can lead to delays in language acquisition and processing, impacting Verbal IQ. Regions like Broca's and Wernicke's areas may develop abnormally, affecting language skills.	Children's brains are in a sensitive developmental phase. Expierence-dependent synapase formation peaks in the early postnatal period where exposure when children are highly susceptible to pollution exposure. This conbination of heightened succpetibility and sensitivity can lead to lasting deficits in both cognitive and language domains.
**Brain Structure and Volume Alteration** 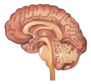	Chronic exposure to $PM_{2.5}$ can lead to structural changes in the brain, such as reductions in gray matter volume or alterations in white matter connectivity. These structural changes can affect regions involved in cognitive and verbal skills (8).	Binter et al. 2022	Structural alterations in brain areas associated with sensory processing (e.g. the parietal lobe) can lead to lower performance IQ, particularly in tasks requiring spatial tasks.	Changes in brain structure, such as reduced volume in language-related centers (e.g. Broca's area), can impact verbal IQ by affecting language processing and production capabilities.	The developing brain of children is more prone to structural damage from pollutants. The ongoing maturation and healthy degredation (i.e. synaptic pruning) of neural circuits makes them more vulnerable to alterations that can impact long-term cognitive and language abilities.

Citations listed provide evidence to support the description of their respective mechanistic pathway. The associated impact on VIQ and PIQ is commentary from the authors of this study.

Figure S1. Human Brain Development: Experience-Dependent Synapse Formation

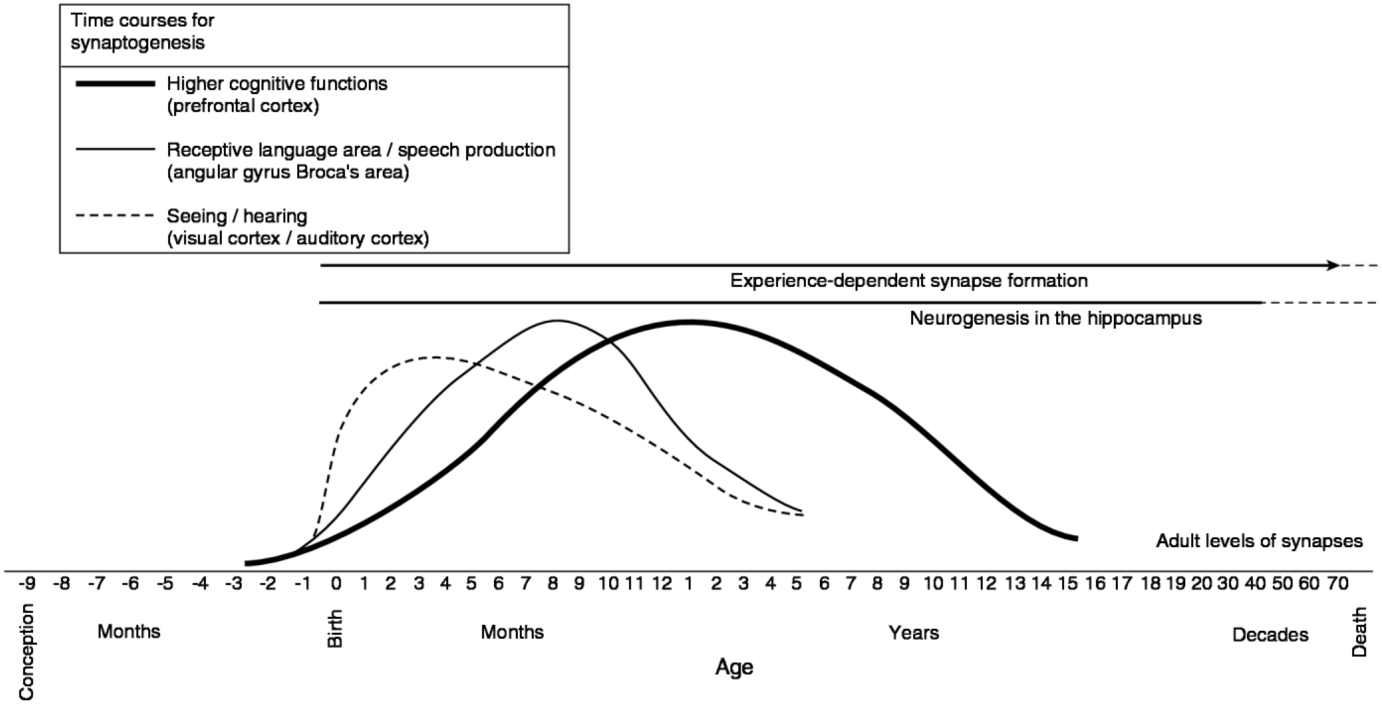

Source: Nelsen, CA, Chapter 8: The Developing Brain “From Neurons to Neighborhoods: The Science of Early Childhood Development”. *National Academies Press.*
https://nap.nationalacademies.org/read/9824/chapter/12

References

1.

1. Sterne JAC, Hernán MA, McAleenan A, Reeves BC, Higgins JPT. Chapter 25: Assessing risk of bias in a non-randomized study [last updated October 2019]. In: Higgins JPT, Thomas J, Chandler J, Cumpston M, Li T, Page MJ, Welch VA (editors). *Cochrane Handbook for Systematic Reviews of Interventions* version 6.5. Cochrane, 2024. Available from www.training.cochrane.org/handbook.
2. Gómez-Budia M, Konttinen H, Saveleva L, Korhonen P, Jalava PI, Kanninen KM, et al. Glial smog: Interplay between air pollution and astrocyte-microglia interactions. Neurochem Int. 2020 Jun;136:104715.
3. Calderón-Garcidueñas L, Calderón-Garcidueñas A, Torres-Jardón R, Avila-Ramírez J, Kulesza RJ, Angiulli AD. Air pollution and your brain: what do you need to know right now. Prim Health Care Res Dev. 2015 Jul;16(4):329–45.
4. Song J, Han K, Wang Y, Qu R, Liu Y, Wang S, et al. Microglial Activation and Oxidative Stress in PM2.5-Induced Neurodegenerative Disorders. Antioxidants (Basel). 2022 Jul 29;11(8):1482.
5. Shang M, Tang M, Xue Y. Neurodevelopmental toxicity induced by airborne particulate matter. J Appl Toxicol. 2023 Jan;43(1):167–85.
6. Gavito-Covarrubias D, Ramírez-Díaz I, Guzmán-Linares J, Limón ID, Manuel-Sánchez DM, Molina-Herrera A, et al. Epigenetic mechanisms of particulate matter exposure: air pollution and hazards on human health. Front Genet. 2024 Jan 17;14:1306600.
7. Han Y, Yu Z, Chen Y, Guo X, Liu Y, Zhang H, et al. PM2.5 induces developmental neurotoxicity in cortical organoids. Environ Pollut. 2024 Sep 6;361:124913.
8. Binter AC, Kusters MSW, van den Dries MA, Alonso L, Lubczyńska MJ, Hoek G, et al. Air pollution, white matter microstructure, and brain volumes: Periods of susceptibility from pregnancy to preadolescence. Environ Pollut. 2022 Nov 15;313:120109.
9. Nelsen C. Chapter 8: The Developing Brain “From Neurons to Neighborhoods: The Science of Early Childhood Development” at NAP.edu. Available from: https://nap.nationalacademies.org/read/9824/chapter/12
